# Study of Cell Behaviors on Anodized TiO_2_ Nanotube Arrays with Coexisting Multi-Size Diameters

**DOI:** 10.1007/s40820-015-0062-4

**Published:** 2015-09-15

**Authors:** Yifan Chen, Jiahua Ni, Hongliu Wu, Ruopeng Zhang, Changli Zhao, Wenzhi Chen, Feiqing Zhang, Shaoxiang Zhang, Xiaonong Zhang

**Affiliations:** 1 State Key Laboratory of Metal Matrix Composites, School of Material Science and Engineering, Shanghai Jiao Tong University, Shanghai, 200240 People’s Republic of China; 2 Suzhou Origin Medical Technology Co. Ltd., Suzhou, 215513 Jiangsu People’s Republic of China

**Keywords:** Coexisting multi-size TiO_2_ nanotubes, Repeated anodization, MC3T3-E1 cells, Cell adhesion behaviors, Cell-material interaction

## Abstract

It has been revealed that the different morphologies of anodized TiO_2_ nanotubes, especially nanotube diameters, triggered different cell behaviors. However, the influence of TiO_2_ nanotubes with coexisting multi-size diameters on cell behaviors is seldom reported. In this work, coexisting four-diameter TiO_2_ nanotube samples, namely, one single substrate with the integration of four different nanotube diameters (60, 150, 250, and 350 nm), were prepared by repeated anodization. The boundaries between two different diameter regions show well-organized structure without obvious difference in height. The adhesion behaviors of MC3T3-E1 cells on the coexisting four-diameter TiO_2_ nanotube arrays were investigated. The results exhibit a significant difference of cell density between smaller diameters (60 and 150 nm) and larger diameters (250 and 350 nm) within 24 h incubation with the coexistence of different diameters, which is totally different from that on the single-diameter TiO_2_ nanotube arrays. The coexistence of four different diameters does not change greatly the cell morphologies compared with the single-diameter nanotubes. The findings in this work are expected to offer further understanding of the interaction between cells and materials.

## Introduction

Numerous studies of novel biomaterials have been revealed to control cell behaviors and direct cell fate, which indicates that material surface properties have profound influence on cell behaviors. However, the complete understanding of the cell-material interaction is still far from clear [1, 2]. A variety of material surface morphologies have been designed to investigate the communication between cells and material surfaces [1, 3, 4]. Recently, TiO_2_ nanotube arrays prepared by anodization have attracted extensive research interests in the medical applications due to their physiochemical properties, such as, high specific surface area, hollow interior structures, super hydrophilicity, interconnected interval among nanotubes, controllable microstructure size, and excellent biocompatibility [5–11]. To date, highly ordered and controlled-structure TiO_2_ nanotube arrays with different structures have been prepared by anodization process, and their effects on cell behaviors have been extensively studied [12–16]. The results of these studies showed that TiO_2_ nanotube arrays substantially enhanced cell adhesion, propagation, differentiation, and mineralization [17–24].

However, many controversies were aroused over the influence of TiO_2_ nanotube arrays on cell behaviors. Schmuki et al. studied the effect of TiO_2_ nanotubes with diameters of 15, 20, 30, 50, 70, and 100 nm on rat mesenchymal stem cell behaviors and reported that 15 nm was the optimal length scale of nanotubes for cell adhesion, propagation, mobility, and differentiation. Meanwhile, the cell adhesion and propagation rate decreased with the increase of nanotube diameters [17, 18]. Jin et al. investigated the influence of TiO_2_ nanotubes with diameters of 30, 50, 70, and 100 nm annealed 2 h on cell behaviors of MC3T3-E1 osteoblasts and hBMSCs, and it was observed that the 30-nm nanotubes could remarkably improve the adhesion of MC3T3-E1 and hBMSCs without obvious differentiation. Also, apparent cell elongation and the highest alkaline phosphatase activity were achieved on 70- and 100-nm nanotubes. The results suggested that the MC3T3-E1 had better osteogenic abilities on 70- and 100-nm nanotubes compared with that on 30-nm nanotubes, and the 70- and 100-nm nanotubes promoted the differentiation of hBMSCs into osteoblasts [19, 20]. Zhang et al. implanted TiO_2_ nanotube arrays with diameters of 30, 70, and 100 nm into mini pigs and revealed that 70-nm nanotubes were the optimum scale for bone conduction and integration [25]. Moreover, our previous research investigated the cell behaviors of MC3T3-E1 on large-diameter TiO_2_ nanotube (150–470 nm). The highest cell elongation (nearly 10:1), the lowest cell number, and the peak of ALP activity were observed on TiO_2_ nanotubes with 470-nm diameter, and the lowest cell elongation and highest cell number were achieved on TiO_2_ nanotubes with 150-nm diameter [26, 27]. We notice that these studies mentioned above were carried out using uniform-diameter TiO_2_ nanotube arrays on the single sample. When the cells were cultured on the uniform-diameter TiO_2_ nanotube arrays, they adhered on this sample without other choices. It is necessary to investigate the cell behavior expression on TiO_2_ nanotube arrays with coexisting multi-size diameters. As we all know, few studies refer to the cell responses to the coexisting multi-size nanostructures.

In the present paper, TiO_2_ nanotube arrays with four different diameters ranging from 60 to 350 nm on the same piece of substrate were prepared by repeated anodization. And then MC3T3-E1 cells were cultured on these samples with coexisting four-diameter TiO_2_ nanotube arrays, and the superiority of cell adhesion was observed. Here we report an apparent inclination of cell adhesion on coexisting different diameters. Our research will be expected to provide inspirations and references for further studies on cell behaviors at initial stage of culture on coexisting multi-size nanostructures.

## Materials and Methods

### Fabrication of Coexisting Four-diameter TiO_2_ Nanotube Arrays

**Fig. 1:**
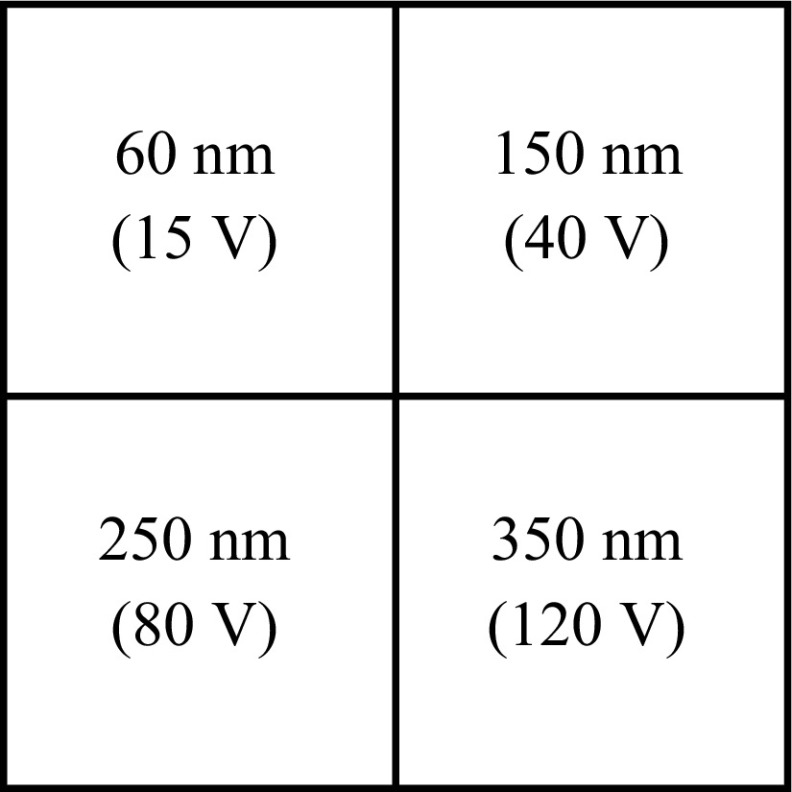
Schematic diagrams of four-diameter coexisting TiO_2_ nanotube arrays

The annealed cp-Ti sheets with 0.25-mm thickness (99.7 % purity, Sigma-Aldrich) were used as starting materials in this research. Before the anodization process, Ti sheets were cut into 2 cm × 2 cm size. These samples were degreased in acetone and then chemically etched for 1 min in the mixture of HNO_3_ and HF to remove the original oxide film. Paraffin wax heated up to above 60 °C was used as paint to cover 3/4 of the sample region, exposing the rest 1 cm × 1 cm region to the electrolyte for anodization. The specimen was anodized using a two-electrode preparation system with a DC power supply (PSB-2400H, GwINSTEK). A platinum electrode served as the cathode. The first 1/4 region was anodized at 15 V for 5 h and then was washed in acetone and chloroform to remove the wax cover. Another 3 steps of anodization were followed then, applying the voltage of 40, 80, and 120 V successively, each for 3 h. The schematic diagrams of the coexisting four-diameter TiO_2_ nanotube arrays are shown in Fig. 1. After anodization, the sample was immersed in acetone for 3 h and then washed with chloroform to remove the leftover wax thoroughly. And then, the sample was rinsed by deionized water followed by a gentle ultrasonication, and dried by air. All the samples used for biological experiments in the following steps were sterilized by dry heat at 160 °C for 2 h.

### Characterization of Surface Topography

A field emission scanning electron microscope (FE-SEM, Quanta 250 FEG, FEI) was utilized to observe the surface morphology and to measure the diameters of coexisting four-diameter TiO_2_ nanotube arrays.

### Contact Angle Measurement

As per the request of contact angle measurement, an optical contact angle measuring device (OCA20, Dataphysics) was utilized to determine water contact angles of the surface of each region of coexisting four-diameter TiO_2_ nanotube arrays.

### Cell Culture

MC3T3-E1 mouse osteoblasts (ATCC CRL-2594, the cell bank of Chinese Academy of Science) were used in this work. Each 1-ml original cells was mixed with 6-ml alpha minimum essential medium (α-MEM, Gibco) in the presence of 10 vol% fetal bovine serum (FBS, Gibco) and 1 vol% penicillin–streptomycin–neomycin antibiotic mixture (PSN, Gibco). Then the cell suspension was plated in a cell culture flask (430639, Corning Incorporated) and incubated at 37 °C in a humidified atmosphere of 5 vol% CO_2_ environment. After 4-day culture, the concentration of MC3T3-E1 cells reached ~3 × 10^5^ cells mL^−1^. A dilution process was carried out to set the concentration to 5000 cells ml^−1^. The cells in the form of cell suspension (4 mL per well) were seeded onto the experimental samples, which were placed on a 6-well polystyrene plate, and incubated at 37 °C in a humidified atmosphere of 5 vol% CO_2_ environment for 2, 6, 12, and 24 h to observe the cell morphology and count the number of viable cells attached at different incubation time. The concentration of the cell seeded onto the specimen substrate was 2 × 10^4^ cells per well.

### Cell Morphology Via SEM

After 2, 6, 12, and 24 h of incubation, the adhered cells on experimental samples were washed with 1 × PBS and fixed with 2.5 % w/v glutaraldehyde (Sinopharm Chemical Reagent Co., Ltd) in 1 × PBS for 4 h. Then, the samples were washed with 1 × PBS and then dehydrated in a gradient series of ethanol (20, 50, 70, 80, 85, 90, 95, and 100 vol%) for 3 min. A critical point dryer (EM CPD300, Leica) was utilized to dry the cells on samples. Finally, the samples with cells were sputter coated with gold by a sputter coater (EMSCD050, Leica) and then were observed via a field emission scanning electron microscope (FE-SEM, Quanta 250 FEG, FEI).

### Fluorescence Observation

In order to observe the distribution of viable cells and count the number of viable cells on the coexisting four-diameter TiO_2_ nanotube arrays, a fluorescein diacetate (FDA, MP Biomedicals) staining was conducted. After the 2, 6, 12, and 24 h incubation process, respectively, the samples with attached cells were washed with PBS. Each 5 mg FDA stock was dissolved in 1 ml acetone and mixed with PBS (10 μL/10 mL). After being incubated in the solution for 30 s and washed again with 1 × PBS, all samples were inverted onto coverslips, visualized, and photographed via a fluorescence microscope (Scope.A1, ZEISS) with a green filter. The fluorescence effect was excited by blue laser.

In order to count the cells adhered on different regions of coexisting four-diameter TiO_2_ nanotube arrays, we established a rectangle coordinate system to cover the whole sample. Each fluorescence picture was taken at the intercross point of the gridding. The software Image Pro Plus, which is specialized in fluorescent cell counting, was applied to calculate the number of viable cells in each picture as the cell density at the intercross point. The distribution of cell density on the whole coexisting four-diameter sample can be expressed by heat map. Fluorescent images of the osteoblast cells cultured 2, 6, 12, and 24 h on coexisting four-diameter TiO_2_ nanotube arrays were taken.

### Statistical Analysis

All experiments were carried out with 4 replicates. SPSS (IBM) was utilized to conduct the statistical significance analysis. The statistical differences were compared by one-way ANOVA analysis and defined as *p* < 0.05. All data were shown as means ± standard errors.

## Results and Discussion

### Morphology of Coexisting Four-diameter TiO_2_ Nanotube Arrays

**Fig. 2:**
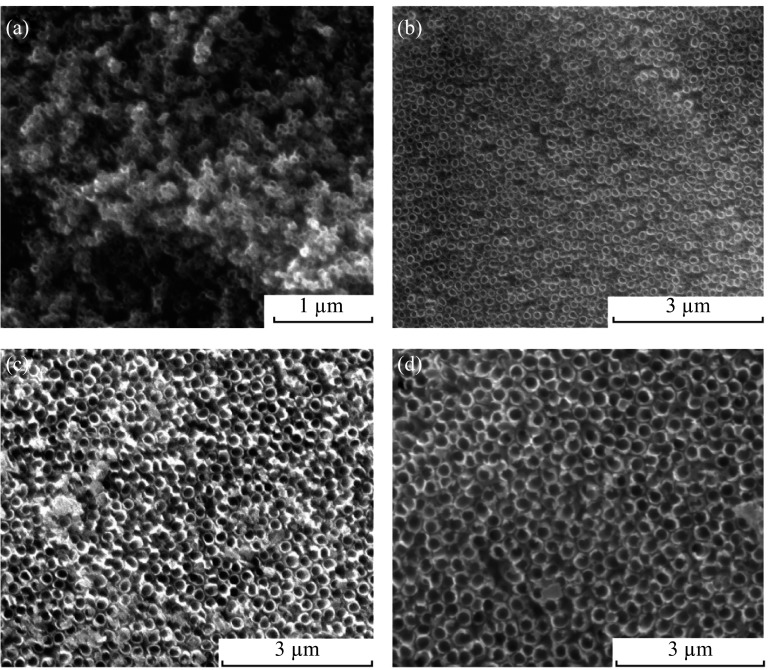
SEM surface morphologies of each region of coexisting four-diameter TiO_2_ nanotube arrays anodized at **a** 60 V; **b** 150 V; **c** 250 V; **d** 350 V

**Fig. 3:**
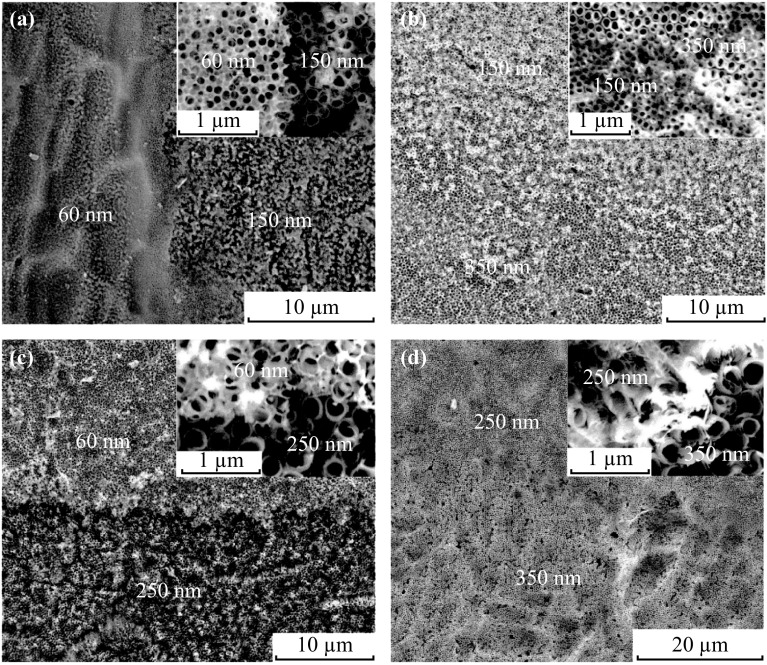
The boundary morphologies between two different diameters of coexisting four-diameter TiO_2_ nanotube arrays: **a** 60 and 150 nm; **b** 150 and 250 nm, **c** 60 and 350 nm, **d** 250 and 350 nm. (All *insets* show partial enlargement of boundary morphologies)

Figure 2 displays the surface morphologies of each region of the coexisting four-diameter highly ordered TiO_2_ nanotube arrays in one single sample. The TiO_2_ nanotube diameters of four regions are 60, 150, 250, and 350 nm, respectively, which were achieved via successively anodization at 15, 40, 80, and 120 V. The boundary morphologies between two different diameters of coexisting four-diameter TiO_2_ nanotube arrays are shown in Fig. 3. From Fig. 3, it is evident that the TiO_2_ nanotubes are also regular at the boundaries between two different regions, and there is no obvious difference in height between two different regions.

### Cell Morphology

**Fig. 4:**
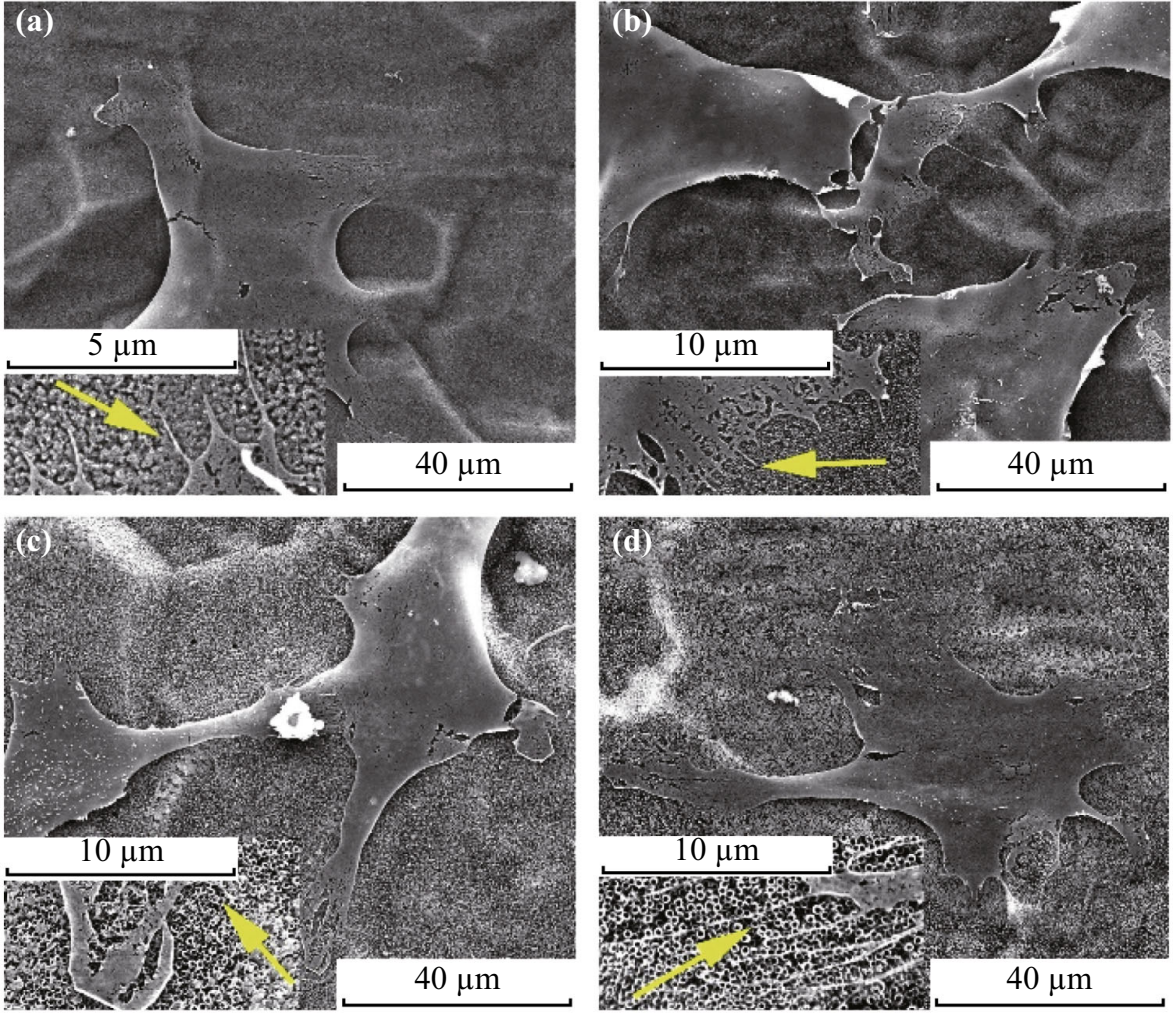
SEM images of cell morphologies after 6 h of incubation on nanotubes of: **a** 60 nm; **b** 150 nm; **c** 250 nm; **d** 350 nm (All *insets* show the filopodia, and the *arrows* indicate the protrusion of filopodia)

Figure 4 shows the morphologies of osteoblasts on different regions. The cells are in round shape on 60-nm-diameter nanotube regions (Fig. 4a), and small amounts of short filopodia could be observed, as shown in the inset in Fig. 4a. With the enlargement of the nanotubes, the cell shape tends to elongate (Fig. 4b, c) and the filopodia are still obvious (inserts in Fig. 4b, c). Osteoblasts sprawl thoroughly on 350-nm-diameter nanotubes (Fig. 4d) and display a large number of long filopodia (inset in Fig. 4d). The changing trend of cell morphologies with nanotube diameter increasing is in agreement with the previous studies [19, 26], which indicates that the combination of four different diameters does not influence the cell morphology, comparing with the single-size nanotube samples.

### Cell Distribution and Counting

**Fig. 5:**
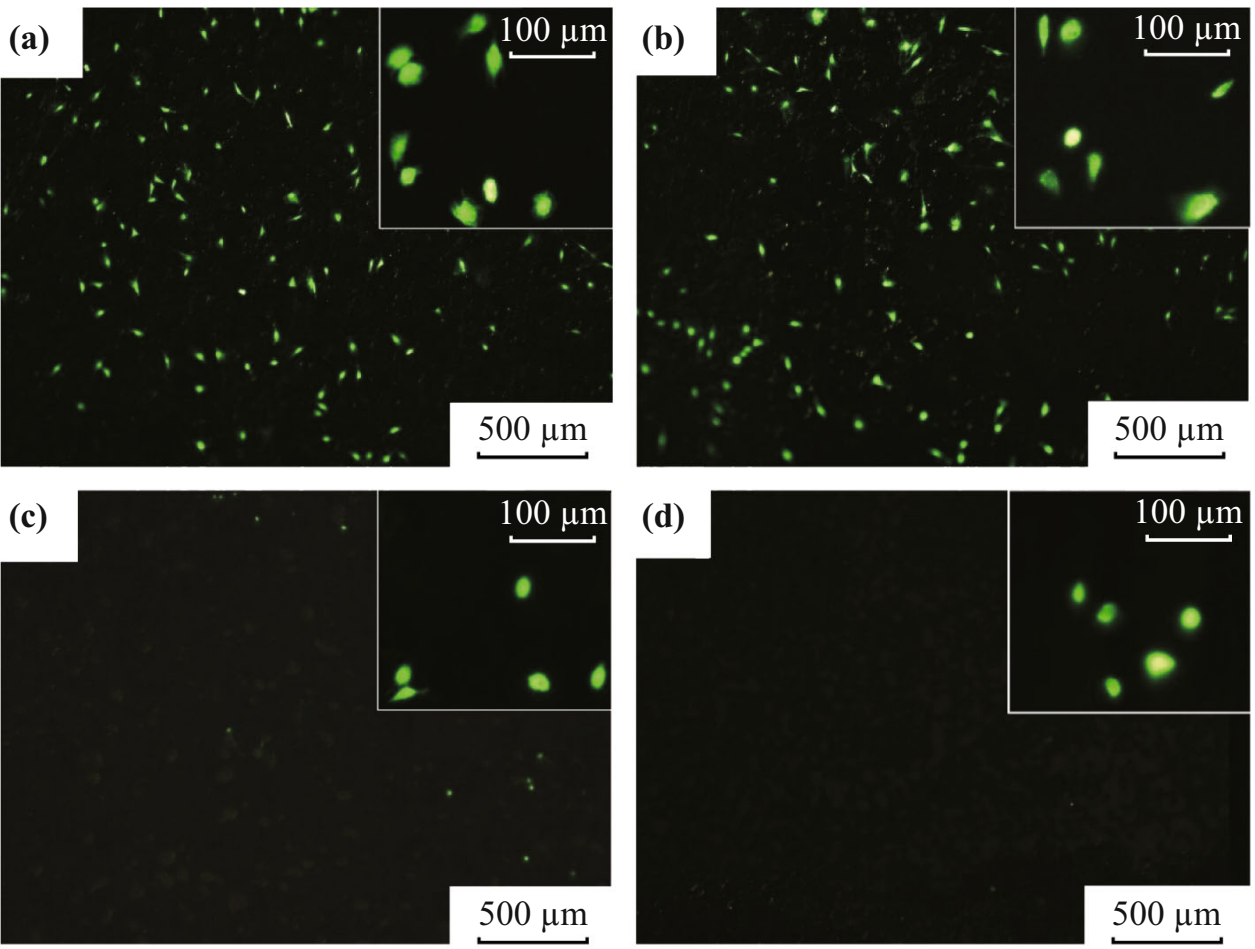
Representative fluorescence images of osteoblasts on each region of coexisting four-diameter TiO_2_ nanotube arrays after incubation of 2 h: **a** 60 nm, **b** 150 nm, **c** 250 nm, **d** 350 nm (All *insets* show the enlargement of representative fluorescence images of osteoblasts)

**Fig. 6:**
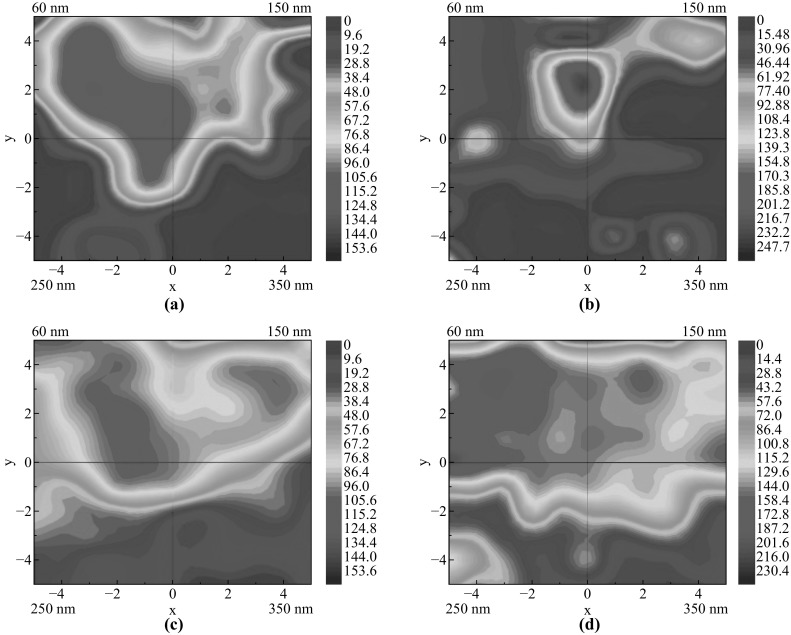
Heat map of cell density distribution on coexisting four-diameter TiO_2_ nanotube arrays after incubation of: **a** 2 h; **b** 6 h; **c** 12 h; **d** 24 h (*Red* shows the highest cell density; *Purple* shows the lowest cell density). (Color figure online)

**Fig. 7:**
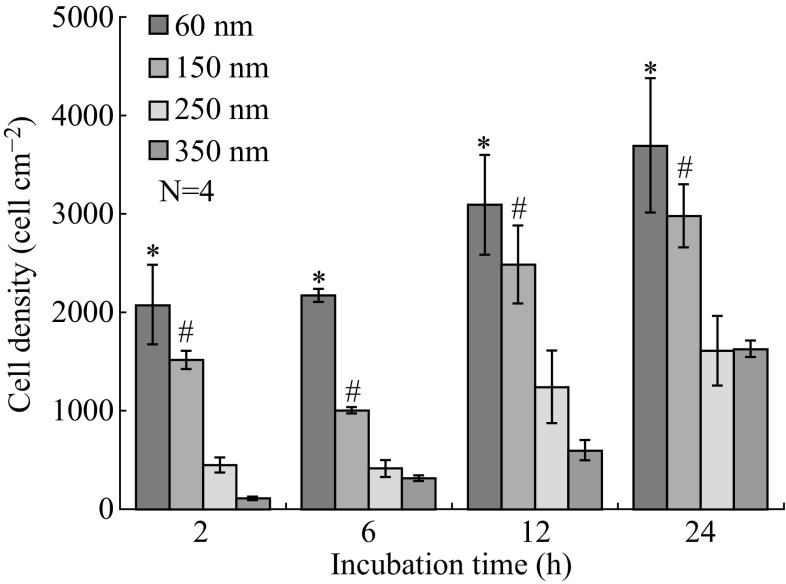
The cell density of each region of coexisting four-diameter TiO_2_ nanotube arrays after incubation of 2, 6, 12, and 24 h. The *bar graphs* show the average ± SD. The *p* values after performing *t* tests reaching statistical significance (*p* < 0.05) are marked on the graphs: *Significant difference between the 60-nm-diameter TiO_2_ nanotubes and the 250- and 350-nm nanotubes; ^#^Significant difference between the 150, 250, and 350 nm in diameters. (Color figure online)

Figure 5 shows the representative fluorescent images of the MC3T3-E1 osteoblasts on each region of coexisting four-diameter TiO_2_ nanotube array after incubation of 2 h. The heat maps (see Fig. 6) illustrate the cell density distribution after incubation of 2, 6, 12, and 24 h on coexisting four-diameter TiO_2_ nanotube arrays, respectively, and Fig. 7 exhibits the total cell density on each region of coexisting four-diameter TiO_2_ nanotubes array after incubation of 2, 6, 12, and 24 h. After 2 h incubation, the highest cell density appears on 60-nm diameter, with the next to be 150 nm in diameter, and the lowest cell density area is 350 nm in diameter (Figs. 6a, 7). The cell counting result shows that 60-nm-diameter nanotubes are 19 times the cell density value of 350-nm-diameter nanotubes, and 5 times more than 250-nm-diameter nanotubes, and 1.5 times more than 150-nm-diameter nanotubes at 2 h of incubation. There is no significant distinction of cell density distribution existing among 2, 6, 12, and 24 h incubation. It is the general tendency that the cell density of viable cells on the smallest diameter nanotubes (60 nm) is higher than other larger ones (150, 250, and 350 nm), and the cell density keeps declining with the increase of TiO_2_ nanotube diameters at the same incubation time, and the 350-nm-diameter nanotubes have the lowest cell density after 2, 6, 12, and 24 h incubation, as seen in Figs. 6 and 7. Also, the 60-nm diameter is 7 times the cell density of 350-nm diameter, and 5 times more than 250-nm nanotubes, and 2 times more than 150-nm nanotubes at 6 h incubation. For 12 h incubation, 60-nm nanotubes are 5 times the cell density of 350-nm nanotubes, and 2.5 times more than 250-nm nanotubes, and 1.3 times more than 150-nm nanotubes. At 24 h, the cell density on 60 nm is 1.3 times more than 150-nm nanotubes, and the cell density on 60 nm is 2.3 times as more as 250-nm and 350-nm nanotubes, and the cell density on 350 nm gets close to 250-nm nanotubes. The cell density on the same region of coexisting four-diameter TiO_2_ nanotubes keeps rising with the extension of incubation time.

The results of the cell density as a function of incubation time reveal that there is a significant difference between 60-nm and larger diameter nanotube (250 and 350 nm), and a significant difference also exists between 150-nm and larger diameter nanotube (250 and 350 nm). However, there is no difference between 250 and 350 nm in the 24 h of incubation, and no difference appears between 60 and 150 nm except 6 h incubation. In this work, the cell suspension covered the whole samples at the very earliest stage of cell suspension ejecting onto the samples, and the cell distribution on the whole samples should have been even without the effect of the special structure of the used sample on cell behaviors. However, the result from fluorescence testing and cell counting (in Figs. 5, 6, 7) shows that the difference of cell density on the regions with different diameters is significant. In our previous study, the MC3T3-E1 cells were cultured on the several groups of single-diameter TiO_2_ nanotube samples with 150–470 nm in diameter, and there was no significant difference of cell density among these single-diameter TiO_2_ nanotube samples at the same incubation time [26], which is totally different from the results in this work. It is concluded that the smaller diameter (less than 150 nm in this work) nanotubes attracted a far greater cell adhesion than larger nanotubes (more than 250 nm in this work) under the coexistence situation of different nanotube diameters. It can be speculated that coexistence of different diameter TiO_2_ nanotubes can promote the cell adhesion on the small diameter TiO_2_ nanotubes at the early stage of cell adhesion, and the smaller diameter nanotubes scramble the chances of cell adhesion from bigger diameter nanotubes. We believe that the phenomenon is attributed to the distinct surface physicochemical properties caused by different diameters.

Previous researches have shown that the surface properties had significant influences on cell adhesion and growth [5, 21]. Ishizaki et al. [28] reported the cells on superhydrophilic surface even started proliferation as soon as they completed the adhesion, and this phenomenon was closely related with the high amounts of the protein adsorption on the hydrophilic surface. Brammer et al. [19] showed that the contact angle of 50–100-nm-diameter TiO_2_ nanotube arrays was about 4–9°, and our previous study [26] showed that contact angle of 150–470-nm-diameter TiO_2_ nanotube arrays ranged from 3 to 7°. The water contact angle of the surface of each region of coexisting four-diameter TiO_2_ nanotube arrays is also about 3–7° (as shown in Table 1). We consider that the wettability difference of 60–350-nm-diameter nanotubes used in this work is not evident, so the wettability is not the main factor which decides the preference of cell adhesion on the smallest diameter nanotubes at the initial stage of cell suspension injection. During the incubation process, the sample is immersed in the media which contain the essential proteins for cell adhesion including fibronectin and albumin. Those relevant proteins are approximately 30–60 nm in diameters, and the initial adsorption of extracellular matrix (ECM) proteins plays a vital role in cell adhesion and its later growth [20, 29, 30]. Therefore, we believe that the small diameter nanotubes provide more anchor points for (ECM) proteins attachment due to higher surface-to-volume ratio compared with larger diameter nanotubes, which leads to more cells adhering on small diameter nanotubes. However, further studies on the influence of small diameters on the cell adhesion under the coexistence situation of different diameter are expected.

**Table 1:** Water contact angle of the surface of each region of coexisting four-diameter TiO_2_ nanotube arrays

Regions	Contact angle (°)
60 nm (15 V)	7 ± 1
150 nm (40 V)	4 ± 1
250 nm (80 V)	3 ± 1
350 nm (120 V)	3 ± 1

## Conclusions

The coexisting four-diameter TiO_2_ nanotube arrays were fabricated via a repeated anodization process at different voltages. The boundaries between two different diameter regions of coexisting four-diameter TiO_2_ nanotube arrays are regular, and there is no obvious difference in height between two different diameter regions. The cell adhesion behaviors of MC3T3-E1 on each region of coexisting four-diameter TiO_2_ nanotube were investigated. The combination of four different diameters does not bring any changes in cell morphologies compared with the single-diameter TiO_2_ nanotubes. The significant difference of cells density exists between the smaller nanotubes (60 and 150 nm) and the larger ones (250 and 350 nm) at 2, 6, 12, and 24 h of incubation, which indicates smaller diameter TiO_2_ nanotubes offer the better triggering structure for cells adhesion than the larger diameter nanotubes under the coexistence situation of different diameter. A further study is needed to investigate the advantage of the smaller diameter nanotubes compared with the larger diameter nanotube. This discovery is expected to give further cognition of cell–material interaction.
